# Sheng-Di-Da-Huang Decoction Inhibited Inflammation Expressed in Microglia after Intracerebral Hemorrhage in Rats

**DOI:** 10.1155/2018/6470534

**Published:** 2018-10-18

**Authors:** Min Cai, Zhonghai Yu, Wen Zhang, Li Yang, Jun Xiang, Jingsi Zhang, Zhennian Zhang, Ting Wu, Xiangting Li, Maodong Fu, Xuxia Bao, Xiaofei Yu, Dingfang Cai

**Affiliations:** ^1^Department of Integrative Medicine, Zhongshan Hospital and Laboratory of Neurology, Institute of Integrative Medicine, Fudan University, Shanghai 200032, China; ^2^Department of Radiology, Shanghai Institute of Medical Imaging, Zhongshan Hospital, Fudan University, Shanghai 200032, China; ^3^Department of Neurology, Nanjing Hospital of Traditional Chinese Medicine/Third Affiliated Hospital of Nanjing University of Traditional Chinese Medicine, Nanjing, Jiangsu Province 210029, China; ^4^Neurology Department, Shuguang Hospital Affiliated to Shanghai University of Traditional Chinese Medicine, Shanghai 201203, China

## Abstract

**Objects:**

Sheng-Di-Da-Huang Decoction was used as an effective hemostatic agent in ancient China. However, its therapeutic mechanism is still not clear. Inflammatory injury plays a critical role in ICH-induced secondary brain injury. After hemolysis, hematoma components are released, inducing microglial activation via TLR4, which initiates the activation of transcription factors (such as NF-*κ*B) to regulate expression of proinflammatory cytokine genes. This study aimed to verify the anti-inflammatory effects of Sheng-Di-Da-Huang Decoction on ICH rats.

**Materials and Methods:**

Intracerebral hemorrhage was induced by injection of bacterial collagenase (0.2 U) in rats. Neurological deficits, brain water content, Evans blue extravasation, expression of TLR4, NF-*κ*B, Iba-1 positive cells (activated microglia), tumor necrosis factor-*α* (TNF-*α*), and interleukin-1*β* (IL-1*β*) were examined 1, 3, 7, and 14 days after collagenase injection. MR images were also studied.

**Results:**

Sheng-Di-Da-Huang Decoction remarkably improved neurological function, reduced brain water content as well as Evans blue extravasation, downregulated expression of TLR4, NF-*κ*B, TNF-*α*, and IL-1*β*, and inhibited microglial activation.

**Conclusions:**

Sheng-Di-Da-Huang Decoction reduced inflammation reaction after ICH through inhibited inflammation expressed in microglia.

## 1. Introduction

Spontaneous, nontraumatic intracerebral hemorrhage (ICH) remains a significant cause of morbidity and mortality throughout the world. Few pharmaceutical therapies have been proved effective in clinical ICH [1]. Since there are no conclusive benefits in clinical trials for primary injury, ICH-induced secondary injury is now the focus of research. The secondary injury of ICH is a dependent progression of 3 intertwined perihematomal degenerative cascade: inflammation, red cell lysis, and thrombin production (coagulation cascade) [2]. Inflammation initiated by microglia is an important part in the cascade. After hemolysis, hemoglobin (Hb) and heme are released from RBCs. Heme induces microglial activation via TLR4, which initiates the activation of transcription factors (such as NF-*κ*B) that regulate expression of proinflammatory cytokine genes. Fibrinogen also activates microglia via TLR4 [3]. Activation of NF-kB in microglia/macrophages after ICH leads to upregulation of proinflammatory cytokines such as TNF-a and IL- 1b and contributes to brain injury [4, 5]. This leads to blood-brain barrier disruption, resulting in cerebral edema and death of brain parenchymal cells [6].

Bacterial collagenase injection into the brain may destroy the extracellular matrix surrounding the blood vessels, which causes cerebral hemorrhage with inflammation, destruction of blood-brain barrier, edema, and tissue necrosis [7]. It can produce highly reproducible cerebral hemorrhage models.

Sheng-Di-Da-Huang Decoction (SDDHD), one of Traditional Chinese Medicine classics, was firstly recorded as “having a magic therapeutic effective on various of hemorrhagic diseases” in “Qian Jin Yi Fang” (also called “Supplement to Invaluable Prescriptions for Ready Reference” or “Supplement to Thousand Golden Prescriptions”). It consists of 2 crude drugs,* Rehmannia glutinosa Libosch*.*, root and rhizome, crude *(Chinese pinyin: sheng di huang, English name:* Rehmannia glutinosa*) and* Rheum officinale Bail., root and rhizome, crude* (Chinese pinyin: sheng da huang, English name:* Rheum officinale*). Both of them are traditionally used as hemostatic agents. It has a significant effect on the neuroprotective effect of hemorrhagic stroke patients and ICH rats by improving the functional activity of nerve cells and promoting their repair [8].

In this study, we focused on the neuroprotective effects of Sheng-Di-Da-Huang Decoction on inflammation reaction expressed in microglia after ICH.

## 2. Materials and Methods

### 2.1. Intracerebral Hemorrhage Model

Male Sprague-Dawley rats weighing 300-350mg were handled and cared for in accordance with the guidelines of the Animal Care and Use Committee (ACUC) of Fudan University and consistent with the National Institutes of Health Guide for the Care and Use of Laboratory Animals. Rats were anesthetized with 10% chloral hydrate (350 mg/kg) and placed in a stereotaxic frame (David Kopf Instruments, Tujunga, CA). A 30-gauge needle was inserted through a burr hole into the caudate nucleus (location 3mm lateral to the midline, 0.2mm anterior to bregma, 6mm in depth below the skull). ICH was induced by administration of 1*μ*L saline containing 0.2U of collagenase (type IV; Sigma, St. Louis, MO) over 5 minutes whereas sham-injected animals received 1*μ*L of saline over the same duration. The needle was slowly removed over 5min, the burr hole was sealed with bone wax, the wound was sutured, and the animal was placed in a warm box with free access to food and water [7]. Rectal temperature was maintained at 37±0.5°C throughout the experimental and recovery periods.

### 2.2. Drugs

The preparation of the decoction is similar to that has mentioned before [9]. Sheng-Di-Da-Huang Decoction is composed of two crude drugs,* Rehmannia glutinosa* and* Rheum officinale*, at a ratio of 3:1. The crude drugs were from TCM Pharmacy of Zhongshan Hospital, Fudan University. Drugs were soaked in distilled water, 1:10 (w/v) drug: water, for 12h. After the first decoction for 1h, the suspension was filtered with gauze. Water was added for the second decoction, which lasted 1h, followed by a third decoction for 1h. The filtered and mixed suspension from the three decoctions was collected and centrifuged at 2000 ×g for 20 m to obtain a suspension for the subsequent preparation. Dehydrated alcohol was added slowly with fast agitation until the concentration reached 75% alcohol (v/v). The solution was stirred overnight, then concentrated to a final concentration of 2 g/mL (w/v), and the precipitate was discarded. The alcohol was evaporated simultaneously with a rotary evaporator. Finally, the liquid was autoclaved and stored at −20°C until use.

The common human daily dose of Sheng-Di-Da-Huang Decoction is 40 g/60 kg bodyweight. According to the formula: *d*_rat_ = *d*_human_ × 0.7/0.11, the common dose of Sheng-Di-Da-Huang Decoction in rats should be 4.2 g/kg/day. In general, the drug tolerance of a rat is higher than that of human; we therefore decided 4, 8, and 16 g/kg/day as the low, medium, and high dosages.

### 2.3. Groups

This experiment was divided into 2 steps. The first step aimed to screen the optimal dose of Sheng-Di-Da-Huang Decoction. Rats were divided into five groups: sham operation group (Sham), model group (ICH), high dose group (High, 16g/kg/day), medium dose group (medium, 8g/kg/day), and low dose group (Low, 4g/kg/day). The rats in the Sheng-Di-Da-Huang Decoction treated groups were orally administered the corresponding doses of Sheng-Di-Da-Huang Decoction, while rats in the sham group and model group were given the same volume of normal saline. Drugs and saline were given from the next day after surgery for 14 days. Functional assessment, brain water contents, and blood-brain barrier (BBB) permeability were evaluated in order to select the best dose.

After the optimal dose was decided, rats were randomly divided into 3 groups: sham operation group (Sham), model group (ICH), and Sheng-Di-Da-Huang Decoction treated group (SDDHD). Magnetic Resonance Imaging (MRI), Iba-1 positive cells, and expression of TLR4, NF-*κ*B, TNF-*α*, and IL-1*β* were examined.

### 2.4. Neurobehavioral Function Evaluation

Neurobehavioral deficits were evaluated by two observers 1, 3, 7, and 14 days after ICH.

#### 2.4.1. Modified Neurological Deficit Scores

The modified version of the neurological deficit scores was designed to assess the motor and sensory nerve injury according to the articles [10, 11] (see Table 1).

#### 2.4.2. Corner Turn Test

Rats were allowed to proceed into a 30 degree-corner, which was formed by two 10*∗*20cm cardboards. They could turn either left or right. Times which rats turn to the right side were counted in a total of 20 times. During this process, rats must stand up and turn to one side, horizontal rotation or abdominal rotation only did not count.

### 2.5. Brain Water Content Measurement

Brain water content was measured with the dry-wet weight method [7]. The brains were removed and separated into hemorrhagic and nonhemorrhagic hemispheres after rats were sacrificed under anesthesia. Both hemispheres were immediately weighed to get the wet weight (WW). The dry weight (DW) was obtained after the tissue were placed in an oven at 100°C for 24h. The brain water content was assessed with the following formula: (WW-DW)/WW×100%.

### 2.6. Blood-Brain Barrier Permeability

To evaluate vascular permeability, a quantitative fluorescent detection of extravasated Evans blue dye was used. Briefly, rats were anesthetized, and 2%EB (2ml/kg) were injected through caudal vein over 3 min at each time points after ICH induction. Two hours later, brains were rapidly removed after perfusion with 200mL normal saline and separated into left and right hemispheres. Both hemispheres were weighed and placed in amide solution (1ml/100g brain tissue). Following incubation at 60°C for 24h and centrifugation at 1000r/min for 5min, the supernatant was measured with a luminescence spectrophotometer (*λ*=632nm), EB content was calculated according to the standard curve. The tissue content of Evans blue was quantified from a linear standard curve derived from known amounts of the dye and was normalized to sample weight [12].

### 2.7. MRI Examination

The MRI scans were performed with a 3.0-T MR scanner system (MAGNETOM Verio, Siemens Healthcare, Germany). Rats were positioned prone under anesthesia with the head inside a 4- channel surface coil designed for the mouse. T2-Weighted Imaging (T2WI) and Susceptibility Weighted Imaging (SWI) were acquired at each time point.

The parameters for the T2-TSE sequence were as follows: matrix 192192, FOV 64mm, TR 2210ms, TE 92ms, flip angle of 120°, 2 average, number of slices 20, and slice thick 1.0mm.

SWI sequence was used 3D gradient echo sequence, and the imaging parameters were comprised as follows: matrix 192 x 192, FOV 50mm, TR 32ms, TE 20.0 ms, flip angle of 15°, 2 averages, 20 coronal slices, and slice thickness 1.0mm.

### 2.8. Western Blotting Analysis

Western blotting was used to assess the expression levels of TLR4 and NF-*κ*B after intracerebral hemorrhage. Corpora striata were collected, prepared in lysis buffer, and centrifuged at 13,000 ×g for 5min. Protein concentrations from supernatant were detected using a BCA kit (Beyotime, Haimen, Jiangsu, China). Protein solution, weighing 30mg, was separated by polyacrylamide gel electrophoresis with different concentrations. The gel was then transferred to polyvinylidene fluoride membranes (Millipore, Bedford, MA, USA), blocked for 1h in a 5% solution of skim milk (dissolved in Tris-buffered saline plus 0.1% Tween-20 (TBST)). The membranes were incubated with primary antibodies and monoclonal rabbit anti-TLR4 and anti-NF-*κ*B antibody (all diluted 1 : 1000; Abcam, HK, China), respectively, at 4°C overnight. The membranes were washed with TBST 3×10 min on the second day and then were incubated with the secondary antibody conjugated with horseradish-peroxidase (Beyotime). The targeted antigens were detected by standard chemical luminescence methods (Beyotime) with Fluor Chem FC2 gel imaging system (Alpha Innotech, Santa Clara, CA, USA). The expression of TLR4 and NF-*κ*B was determined by using the GADPH protein as the internal reference. Western blots were duplicated with three independent sets. Band intensities were measured with Image J software.

### 2.9. Immunofluorescence Staining

Immunofluorescence staining was used to evaluate the expression of activated microglia. Rat brains were removed after cardiac perfusion with 200mL normal saline followed by 150mL 4% paraformaldehyde, fixed in 4% paraformaldehyde for 48h. Fixed brains were cut coronally through the needle entry site (identifiable on the brain surface), as well as 2mm anterior and 2mm posterior to that plane. The striatum was cut into 4*μ*m thick each coronary slice. These slices were deparaffinized and incubated with 0.3% H_2_O_2_ in PBS. After blocking with 5% bovine serum albumin (BSA) serum, the sections were incubated with anti-Iba-1 antibody (diluted 1:100; Abcam) at 4°C overnight. On the second day, slices were covered with fluorescent-labeled secondary antibody FITC-conjugated anti-rabbit IgG (1:100, Beyotime), at 37°C for 30min, followed by DAPI (Beyotime) for 10min after washed in PBS. All sections were photographed and observed with a light microscope (Olympus/BX51, Tokyo, Japan).

### 2.10. Enzyme-Linked Immunosorbent Assay

TNF-*α* and IL-1*β* contents in the brain tissue around the hematoma were measured by Enzyme-linked immunosorbent assay (ELASA). The animals were anesthetized with 10% chloral hydrate, the brain was decapitated, and the striatum was removed and weighed. 1ml/100 mg of brain tissue was added to PBS, fully homogenized under ice bath and centrifuged at 12000 ×g for 10min. According to the operation instructions of ELISA kit (Elabscience), standard curves were drawn to calculate the concentration of TNF-*α* and IL-1*β*.

### 2.11. Statistical Analysis

Data analysis was performed with SPSS version 22.0 (SPSS, Chicago, IL, USA). All variables were expressed as means ± standard error of the mean (SEM). Kruskal-Wallis test followed by Mann-Whitney test was used to analyze data of neurological deficit scores, while other data were analyzed with ANOVA and post hoc Bonferroni-Dunn correction for intergroup comparisons after tested homogeneity of variance. Differences were considered statistically significant when* P *< 0.05.

## 3. Results

### 3.1. High Dose of Sheng-Di-Da-Huang Decoction Improved Neurological Function

**Figure 1:**
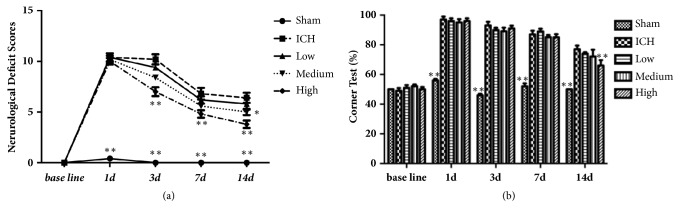
(a) Neurological deficit score (maximum total score is 13) of rats after injection of collagenase. (b) The result of corner turn test. Data were presented as mean ± SEM, n=5 rats for each group. *∗ P*<0.05; *∗∗* <0.01 compared with ICH group.

The success rates of ICH models were approximately 96%. Modified neurological deficit scores from 1 to 14 days after collagenase injection are shown in Figure 1(a). Sham-operated rats had almost no neurological deficit at all time points after sham injection. Rats in other groups were significantly neurological impaired (approximately 10) 1 day after collagenase injection. Improvement was noted from 3 days. Recovery differed between four groups. The scores of high dose group were significantly lower than that of ICH group at 3, 7, and 14 days (P<0.01). Besides, the score of medium dose group was lower than that of ICH group at 14d (P<0.05) (Figure 1(a)).

The baseline of the corner turn test result was about 50%, as the probability of left or right turn was basically equal. When intracerebral hemorrhage occurred in the right side of the brain, motor and sensory function of the left side were all damaged, leading to the probability of right turn increasing significantly in ICH group (almost 100%) compared with sham group (P < 0.01). The ratio in high dose group decreased significantly (P < 0.01) than ICH group, which indicated that neurological function in rats had certain recovery (Figure 1(b)).

### 3.2. Sheng-Di-Da-Huang Decoction Reduced Brain Water Content

**Figure 2:**
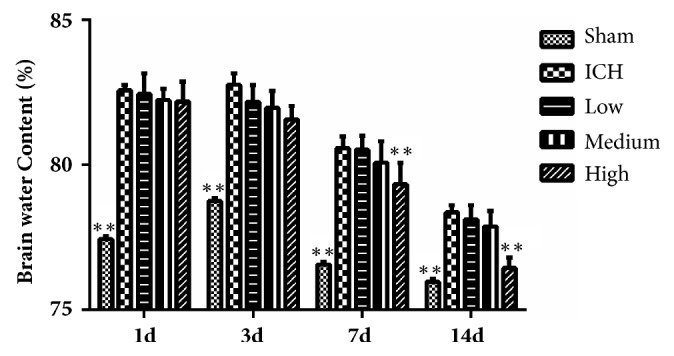
Result of brain water contents for each group at different time points. Data were presented as mean ± SEM, n=5 rats for each group. *∗ P*<0.05; *∗∗* <0.01 compared with ICH group.

Compared with sham group (76-78%), rats with intracerebral hemorrhage had significantly increased brain water content in the damaged striatum from 1 day to 14 days, which was significantly reduced by high dose SDDHD at 7 and 14 days (Figure 2).

### 3.3. Sheng-Di-Da-Huang Decoction Reduced the Permeability of Blood-Brain Barrier

**Figure 3:**
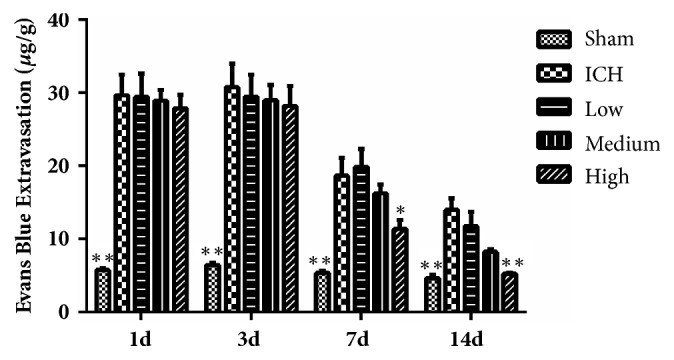
Results of Evans blue extravasation in each group at different time points. Data were presented as mean ± SEM, n=5 rats for each group. *∗ P*<0.05; *∗∗* <0.01 compared with ICH group.

Under normal circumstances, Evans blue cannot pass through the BBB. Bacterial collagenase destroys the cerebral vascular basal lamina, resulting in leakage of blood into the surrounding brain parenchyma, damaging the BBB. In sham group, the needle slightly damaged the BBB, therefore, a small amount of leakage appeared. Evans blue leakage was obvious in ICH group (P < 0.01 compared with the sham group) and significantly reduced by high dose SDDHD at 7 and 14 days, which might suggest that SDDHD could help repair the BBB (Figure 3).

Considering that the neurological deficits are not always synchronous with pathological changes, neurological deficit was not consistent with edema and BBB disruption. Take the results of the neurological function tests, brain water content, BBB permeability, and the adverse effect of higher dose of Sheng-Di-Da-Huang Decoction (severe diarrhea) into account, 16 g/kg/day became the therapeutic dose for the next experiments.

### 3.4. MRI Results

**Figure 4:**
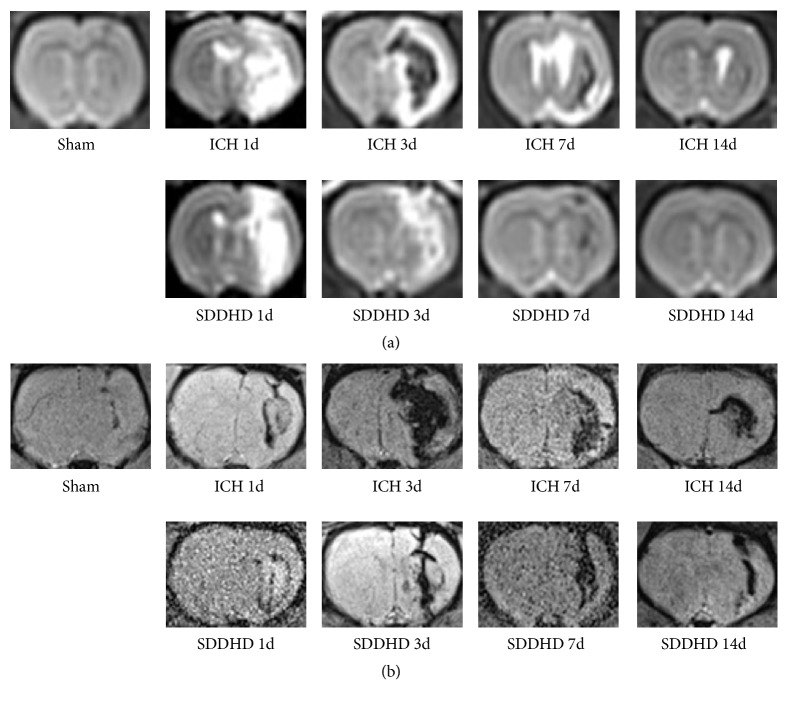
Results of MRI study. The images, taken at the different time points, were from a single rat. (a) Representative T2-weighted MR images of intracerebral hematoma in rat brain at the level of the collagenase injection. (b) Representative SWI images of intracerebral hematoma in rat brains surrounding collagenase injection. n=5 rats for each group at each time point. The images were from different rats in the same group.

T2WI showed the intensity evolution of hematoma at different time points. There was no obvious hyperintensity or hypointensity area on T2WI at 14 days (Figure 4(a)), while on SWI the cerebral hemorrhagic areas were still clearly visible (Figure 4(b)). Hematoma size was calculated based on T2WI. Area of hyperintensity as well as hyperintensity-hypointensity mixed region of 10 coronary images were measured, multiplied by the thickness of the slice 1mm. There was no significant difference between ICH group and SDDHD group (40.85±10.25mm^3^ versus 39.52±12.20mm^3^ at 1 day; 34.58±9.84mm3 versus 32.69±8.35mm3 at 3 days; 21.54±6.27mm3 versus 21.18mm3 at 7 days; 23±2.36mm3 versus 8.95±3.69mm3 at 14 days).

### 3.5. Sheng-Di-Da-Huang Decoction Inhibited the Activation of Microglia

**Figure 5:**
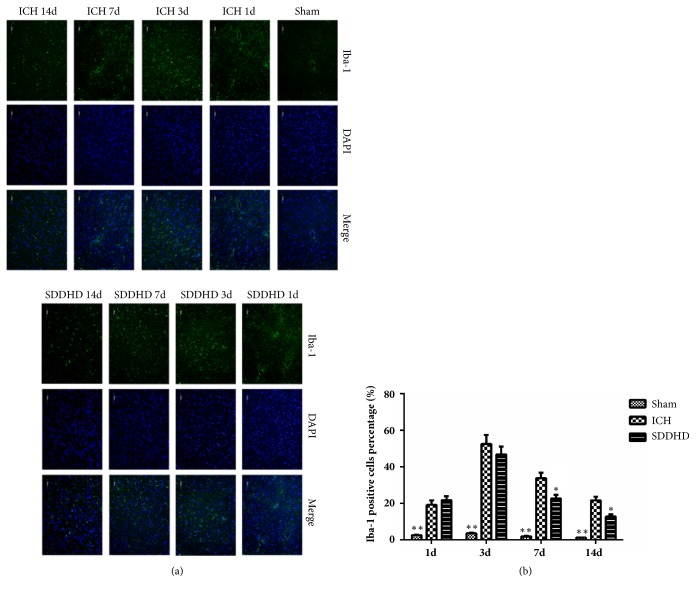
(a) Representative immunofluorescence images of Iba-1, ×200 magnified. (b) Iba-1 positive cells percentage (green fluorescence represents Iba-1 positive cell). Data were presented as mean ± SEM, n=5 rats for each group. *∗ P*<0.05; *∗∗* <0.01 compared with ICH group.

Iba-1 positive cells increased significantly in ICH and SDDHD groups compared with sham group (Figure 5(a)). The Iba-1 cells percentage (over all DAPI positive cells) reached the peak at 3 days, and decreased over time. It significantly decreased in SDDHD group at 7 and 14 days compared with ICH group (*P*<0.05) (Figure 5(b)).

### 3.6. Sheng-Di-Da-Huang Decoction Downregulated the Expression of TLR4, NF-*κ*B, TNF-*α*, and IL-1*β*

**Figure 6:**
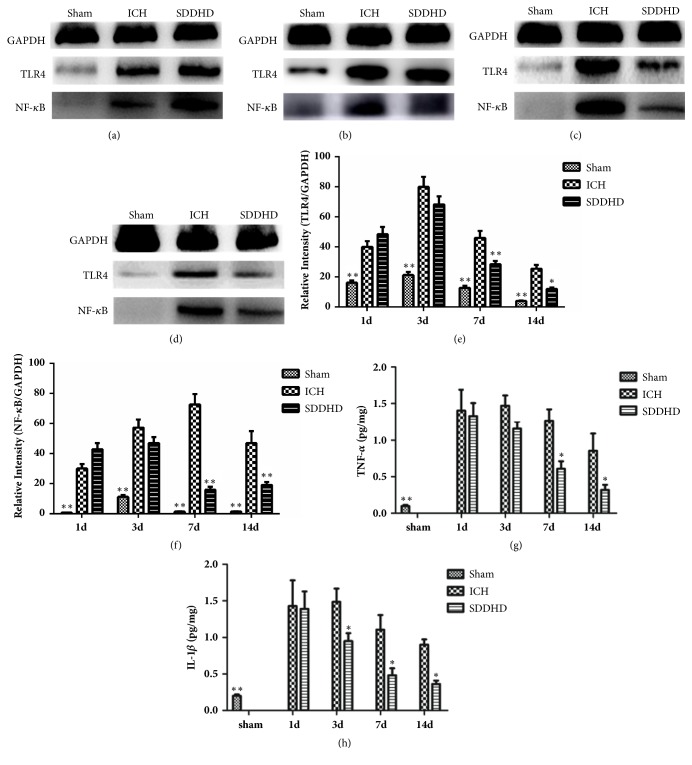
Detection of TLR4 and NF-*κ*B in the hemorrhagic area using Western blotting. (a) The bands of GAPDH, TLR4, and NF-*κ*B at 1 day. (b) The bands of GAPDH, TLR4, and NF-*κ*B at 3 days. (c) The bands of GAPDH, TLR4, and NF-*κ*B at 7 days. (d) The bands of GAPDH, TLR4, and NF-*κ*B at 14 days. (e) Quantitative results of the bands for TLR4 relative to GAPDH at each time point. (f) Quantitative results of the bands for NF-*κ*B relative to GAPDH at each time point. Data are presented as mean ± SEM, n=5 rats for each group. *∗ P*<0.05; *∗∗* <0.01 compared with ICH group. Results of TNF-*α* (g) and IL-1*β* (h) concentrations in each group at different time points. Data were presented as mean ± SEM, n=5 rats for each group. *∗ P*<0.05; *∗∗* <0.01 compared with ICH group*∗*.

WB results showed that, compared with sham group, TLR4 and NF-*κ*B overexpressed in ICH group. Expressions of TLR4 and NF-*κ*B reached peak at 3 days and 7 days, respectively, and were inhibited in SDDHD group at 7 and 14 days (Figures 6(a)–6(f)).

Time course showed the protein levels of TNF-*α* (Figure 6(g)) and IL-1*β* (Figure 6(h)) in the striatum. While there was little TNF-*α* and IL-1*β* in sham group, there were marked expressions in ICH and SDDHD groups. That upregulations significantly increased at 1 day, peaked at 3 days (TNF-*α*) or 7 days (IL-1*β* ), and gradually decreased over two weeks. Concentrations of both TNF-*α* and IL-1*β* were significantly reduced in SDDHD group.

## 4. Discussion

The present study found that Sheng-Di-Da-Huang Decoction could significantly improve neurological function, reduce brain water content, protect blood-brain barrier, and inhibit inflammation reaction after ICH, and the possible mechanism was through inhibiting inflammation expressed in microglia.

Sheng-Di-Da-Huang Decoction is a traditional Chinese prescription and its effectiveness has been proved through hundreds years of clinical verification. It consists of crude* Rehmannia glutinosa* and* Rheum officinale*. More than 140 individual compounds have been isolated from* Rehmannia glutinosa. *Catalpol, ajugol, and acetoside are thought to be its active constituents [13]. It affects hemorheology of hematopoietic system and has hemostatic efficacy. Both crude rehmannia root and rehmannia dried rhizome showed an antagonism of coagulation time prolonged by aspirin, and the effect of crude rehmannia root was stronger [14]. Besides, it has multiple pharmacologic effect on nervous system, including antiinflammatory activity, sedation effect, and apoptosis attenuation [15].* Rheum officinale* also contains a lot of compounds, such as sennosides A–F [16]. As a laxative, it is much more potent than cascara [17]. The most important active substances of* Rheum officinale* are emodin and Rhein. Emodin has multiple pharmacological effects, including changing the ion concentration and transportation, antioxidation, removing free radicals, affecting inflammatory factors secretion, enzyme activity, gene synthesis, and expression [18–21]. Rhein has anti-inflammatory and immunosuppressive effects and can effectively inhibit the activity of NF-*κ*B, leukocyte interleukin 6 (IL-6), nitric oxide (NO), macrophage inflammatory protein-1*β*, and matrix metalloproteinases (MMPs) [22–24].

TLR signaling after intracerebral hemorrhage has been reviewed [25]. After hemolysis, different components of the hematoma activate microglia via distinct pathways. Heme induces microglial activation via TLR4/ NF-*κ*B which regulates expression of proinflammatory cytokine genes. Fibrinogen also activates microglia via TLR4 [3]. TLR4 protein expression is significantly increased within several hours, peaks at three days following ICH, and remains elevated relative to baseline [26–28]. Compared with WT mice, TLR4-knockout mice exhibited less microglial activation after ICH [26, 29], and lower levels of inflammatory cytokines, such as TNF-a, IL-1*β*, and IL-6, as well a corresponding decrease in NF-*κ*B activity [26]. TNF-*α* and IL-1*β*, two major proinflammatory cytokines, play a major role in exacerbating ICH-induced brain injury. Both of them have been shown to be upregulated after ICH in animal models and clinical studies, bringing detrimental effects including brain edema and BBB disruption [4, 30]. The present study showed SDDHD treatment may could inhibit inflammation.

Microglia are the resident macrophages of the brain and protect neuronal function under normal condition. Its role in ICH is bilateral. In one hand, activated microglia clear the hematoma and damaged cell debris through phagocytosis; on the other hand, excessive microglial activation releases a variety of cytokines; free radicals, nitric oxide, and other toxic chemicals thereby aggravate ICH-induced brain injury. They are activated within 1 hour after ICH onset, peaked at 3 days, and lasted for 7 days [31]. Several animal studies have shown the beneficial effects of inhibition of microglial activation. Minocycline, a tetracycline-class antibiotic, is the most studied one. It can attenuate brain edema, BBB leakage, and brain cell death in a rat model of ICH [32, 33]. However, studies show that minocycline has a very narrow therapeutic window (within 3 h of ICH onset) [34, 35], and clinical researches fail to prove its clinical effectiveness. The present study showed Sheng-Di-Da-Huang Decoction treatment could inhibit microglia activation, which indicates one of the possible mechanisms of its neuroprotective effects in ICH.

BBB destruction after ICH is associated with inflammation. Reactive oxygen species, proinflammatory cytokines, chemokines, and matrix metalloproteinases may all damage the BBB, leading to vascular edema. The edema increases intracranial pressure, causing cerebral herniation [36]. In animal model of intracerebral hemorrhage, edema reached its peak at 3-4 days after ICH, and was then gradually absorbed [37, 38]. The present study showed that BBB was damaged and brain edema formed at 1 day. Damage reached the peak at 3 days. Sheng-Di-Da-Huang Decoction could protect BBB and alleviate edema by inhibiting inflammation.

The evolving appearance of human ICH on T_2_-weighted MR images is related to the effects of hemoglobin degeneration and changes of edema. At the center of the hematoma, intensities differed from early (1 to 12 hours) hypointensity to later hyperintensity (1 to 2 days), and strongly hypointense rim around the resolving hematoma after 3 days [7]. In the present study, findings were in accordance with it, and Sheng-Di-Da-Huang Decoction seemed to accelerate the evolving appearance.

In conclusion, this study showed that Sheng-Di-Da-Huang Decoction had a neuroprotective effect on ICH. There may be other mechanisms and cells in addition to microglia are involved. More explicit mechanisms remain to be discovered in further research.

## Figures and Tables

**Table 1:** Modified neurological deficit scores.

Items	Scores
Neurological deficit scores^a^	0-4
Tail test^b^	0-2
Placing test	0-7
Vision^c^	0-1
Tactile sensation^d^	0-6
Forelimbs	0-2
Hindlimbs	0-2
Lateral	0-2

Total	0-13

a: scores were as follows: 0 points, no neurological deficit; 1 point, left forelimb not fully extended; 2 points, circling to the left; 3 points, dumping to the left; 4 points, no spontaneous activity or coma.

b: scores were as follows: 0 points, no flexion; 1 point, the left forelimb flexion or trunk torsion to the right side; 2 points, the left forelimb flexion and trunk torsion to the right side

c: scores were as follows: the tester held the rat face close to the edge of the table, whisker cut short. Normally, rats would reach the desktop by their bilateral forelimbs. 0 points, the rats showed normal; 1 point, left forelimb could not reach the table.

d: scores were as follows: rats were placed on the edge of the table, with forelimbs hanging at the edge of the table and free move. One side of forelimbs were gently pulled down. The tester observed the forelimbs back to the original place and repeated the other side of the forelimbs, using the same method to observe the activities of hindlimbs. Finally, the rats were placed in parallel to the table edge, and the lateral movement of the forelimbs was observed. 0 points, normal stretch (forward, backward, or lateral); 1 point, stretch but more than 2s delay and/or extension not complete; 2 points, no stretch.

## Data Availability

The data used to support the findings of this study are available from the corresponding author upon request.
